# Suture-based fundoplication techniques during peroral endoscopic myotomy

**DOI:** 10.1016/j.vgie.2025.11.015

**Published:** 2025-12-11

**Authors:** Farimah Fayyaz, Michael Bejjani, Romina Roshanshad, Mouen Khashab

**Affiliations:** 1 Gastroenterology and Hepatology Division, Johns Hopkins Medicine, Baltimore, Maryland, USA; 2 Division of Gastroenterology, Department of Medicine, Chulalongkorn University, Bangkok, Thailand

## Abstract

**Background and Aims:**

Peroral endoscopic myotomy (POEM) with fundoplication (POEM-F) combines POEM with endoscopic fundoplication to prevent postprocedural gastroesophageal reflux. The conventional techniques using the loop-ligating device can be technically challenging, with durability issues. Endoscopic suturing may address these limitations. We describe 2 novel suture-based techniques for fundoplication after POEM.

**Methods:**

Two patients with type II achalasia underwent POEM-F using suture-based fundoplication approaches.

**Results:**

In the first case, an over-the-scope suturing system was used to place seromuscular sutures from the peritoneal side between the gastric fundus and the distal myotomy edge, creating a wrap secured with a cinch. In the second, a T-Tag suture (OverStitch 2-0 Polypropylene Suture; Boston Scientific, Marlborough, Mass, USA) was retrieved from the gastric lumen using forceps through a 19-gauge needle from the serosal side. Helical tacks were placed over the externalized suture and anchored to the peritoneum at the distal end of the myotomy. The wrap was completed by cinching the suture, with the T-Tag left in the gastric lumen. No adverse events were observed.

**Conclusions:**

Suture-based techniques for POEM-F offer a promising alternative to loop-ligating methods, potentially improving procedural control, and warrant evaluation of long-term durability. These techniques remain off-label and technically demanding but may be further optimized by the development of dedicated endoscopic suturing systems tailored for POEM-F.

## Introduction

Peroral endoscopic myotomy (POEM) with fundoplication (POEM-F) is a natural-orifice transluminal endoscopic surgery (NOTES)–based approach to prevent gastroesophageal reflux disease (GERD) after POEM.^1^ The traditional technique involves transmural dissection over gastric myotomy to access the peritoneum, where a loop-ligating device is anchored to the gastric fundus and myotomy edge using clips to create a wrap. However, the difficult passage of the loop-ligating device alongside the gastroscope through the submucosal tunnel, sharp edges, and limited maneuverability can complicate deployment and risk mucosal injury.

Suturing offers a potential alternative with improved maneuverability and reduced risk of trauma. Although POEM-F using loop-ligating devices has shown favorable short-term outcomes and safety,^2^^,^^3^ suture-based approaches remain largely theoretical, with only a single case report to date, to our knowledge.^4^ We describe 2 experimental procedures using suture-based techniques for performing fundoplication after POEM.

## Methods

### Standard fundoplication technique

**Figure 1:**
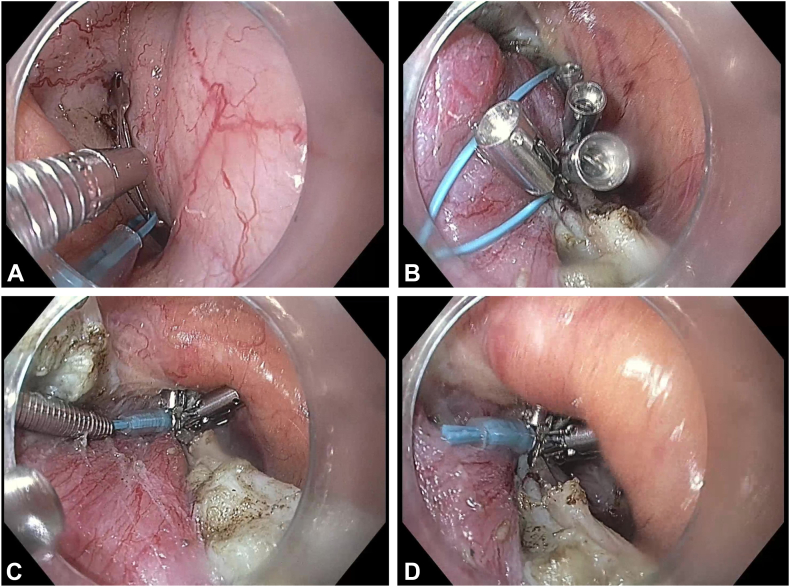
Standard fundoplication technique using endoloop. **A,** Endoloop (PolyLoop; Olympus America, Center Valley, Pa, USA) is secured to the gastric fundus using clips. **B,** Proximal end of the endoloop is fixed to the distal end of the myotomy. **C,** Endoloop is tightened. **D,** Tail is trimmed.

The conventional POEM-F approach uses a loop-ligating device to create a fundoplication wrap, as previously described.^2^ Briefly, after POEM and peritoneal entry, an insufflation needle (Applied Insufflation Needle 13-gauge 120 mm; Applied Medical, Rancho Santa Margarita, Calif, USA) connected to a robotic insufflator (EVA15; Palliare, Inc, Oceanside, Calif, USA) is inserted through the anterior abdominal wall under direct visualization with the intraperitoneal flexible endoscope to maintain intraperitoneal pressure.^5^ This method of intraperitoneal insufflation enhances visualization and working space during the procedure, particularly in patients with significant adipose tissue. Although we do not perform fundoplication in patients with a body mass index greater than 30, we also refrain from creating the wrap if visualization is inadequate, because safe and effective fundoplication requires clear identification and controlled approximation of the fundus. Subsequently, the anterior fundus just distal to the angle of His is retracted into the submucosal tunnel using raptor forceps and under ultraslim endoscope (GIF-XP190N; Olympus America, Center Valley, Pa, USA) visualization with the stomach fully distended. Once the wrap position is confirmed to cover ≥180° of anterior circumference without any torsion, the position on fundic serosa is marked, and the endoloop (PolyLoop; Olympus America) is delivered into the peritoneal cavity alongside the standard gastroscope. Through-the-scope clips secure the loop to both the fundus and the distal myotomy. The loop is then tightened under direct visualization, and the tail is trimmed (Video 1, available online at www.videogie.org, Fig. 1). POEM-F is a technically demanding procedure that requires advanced endoscopic skills and familiarity with NOTES techniques. The lack of dedicated instruments increases the risk of technical failure and adverse events. Based on literature and our own experience, potential risks and technical challenges include mucosal injury, perforation or leak, bleeding, infection, gas-related events, technical failure of the fundoplication wrap, limited fundus visualization, clip erosion, and wrap loosening or partial failure.^2^^,^^6^^,^^7^ The surgical team is kept on standby to perform emergency surgery if needed.

### Novel suture-based fundoplication techniques

Both procedures followed the standard POEM-F technique through peritoneal access and initial wrap simulation. The approach diverged at the point of fundoplication.

### Case 1: over-the-scope suturing

**Figure 2:**
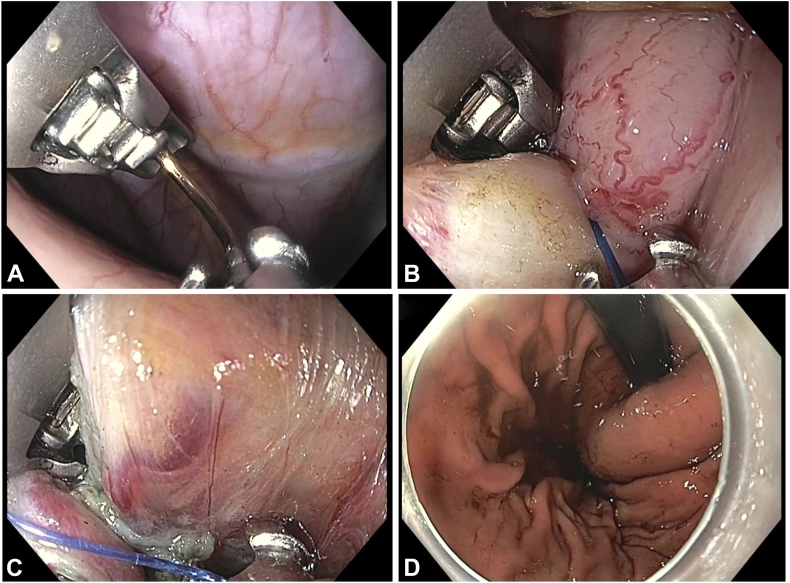
Over-the-scope suturing technique. **A,** Suturing system is advanced into the peritoneal cavity. **B,** First seromuscular bite is taken from the gastric fundus. **C,** Second seromuscular bite is taken from the distal myotomy edge. **D,** Final formed wrap.

A double-channel gastroscope (GIF-HQ190; Olympus America) with an over-the-scope suturing system (OverStitch; Boston Scientific, Marlborough, Mass, USA) was introduced into the submucosal tunnel using a floating technique with the minimum volume of sterilized saline required for device passage. The device was advanced into the peritoneal cavity where 2 seromuscular bites were taken: 1 from the gastric fundus and 1 from the distal myotomy edge. The suture was tightened and cinched, forming an adequate wrap (Fig. 2).

### Case 2: helical anchoring technique with T-Tag suture

**Figure 3:**
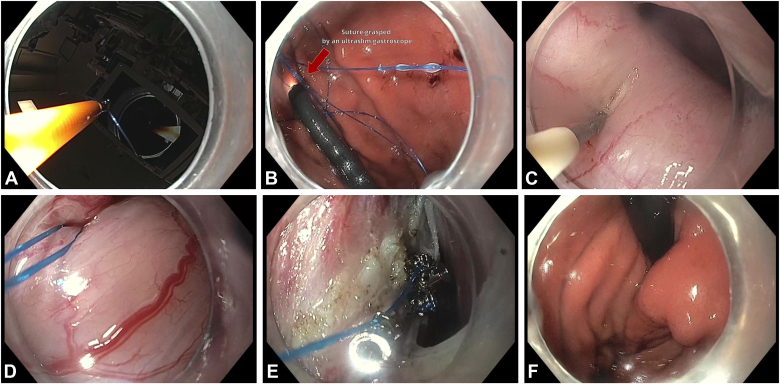
Helical anchoring technique with T-Tag suture (OverStitch 2-0 Polypropylene Suture; Boston Scientific, Marlborough, Mass, USA). **A,** Grasping a suture with a T-Tag using forceps. **B,** The suture is grasped in the stomach by pediatric forceps introduced through an ultraslim scope. **C,** A needle is used to puncture the stomach from the serosal side. **D,** The suture is pulled through the stomach wall into the peritoneal cavity. **E,** Four helical tacks are secured at the distal end of the myotomy in the peritoneum. **F,** Final formed wrap.

A T-Tag suture (OverStitch 2-0 Polypropylene Suture; Boston Scientific) was grasped using forceps (Radial Jaw 4 Large Capacity with Needle; Boston Scientific) through a therapeutic gastroscope (GIF-HQ190) and passed into the stomach. Pediatric forceps (Radial Jaw 4 Pediatric Biopsy Forceps with Needle; Boston Scientific) inserted through an ultraslim scope (GIF-XP190N) grasped the suture inside the stomach. The therapeutic scope was then maneuvered through the tunnel to the peritoneum, and a 19-gauge needle (Expect EUS-FNA Needle; Boston Scientific) was used to puncture the gastric wall from the peritoneal side, and microforceps (Moray Micro Forceps; STERIS, Mentor, Ohio, USA) passed through the needle retrieved the suture into the peritoneum and submucosal tunnel. Four X-Tack HeliX Tacks (Boston Scientific) were placed over the retrieved suture and anchored at the distal end of the myotomy in the peritoneum. The suture was cinched to create the wrap, with the T-Tag left inside the gastric lumen (Fig. 3).

Additional challenges with novel suture-based approaches include difficulty advancing or positioning the device in the tunnel, use of a single suture due to technical constraints, longer procedure time and limited visualization, and the thin distal end of myotomy muscle layer limiting suture strength.

## Results

Case 1 had a procedure time of 84 minutes and a fundoplication time of 62 minutes (Table 1). At the 15-week follow-up, the Eckardt score improved from 10 to 0, with no reflux symptoms. Endoscopy showed an intact wrap and grade A esophagitis; pH monitoring was normal (DeMeester score 1.5, acid exposure 0.4%).

**Table 1:** Procedural summary and GERD outcomes of 2 novel POEM-F techniques

Parameter	Case 1	Case 2
Technique name	Over-the-scope suturing	Helical anchoring technique with T-Tag suture (OverStitch 2-0 Polypropylene Suture; Boston Scientific, Marlborough, Mass, USA).
Technique summary	• Over-the-scope suturing device entry into tunnel using floating technique. • Entry into the peritoneum. • Two seromuscular bites (gastric fundus, distal myotomy edge). • Suture tightened and cinched to create the wrap.	• Dual-scope relay of T-Tag suture into the stomach. • Peritoneal puncture with 19-gauge endoscopic ultrasound needle; microforceps retrieve suture into the peritoneum/tunnel. • Four tacks anchor at distal myotomy; suture cinched to form wrap; T-Tag left in the gastric lumen.
Fundoplication time, min	62	119
LA grade esophagitis (3 mo)	Grade A esophagitis	Grade B esophagitis
DeMeester score (3 mo)	Normal (1.5)	Normal (9.0)
Acid exposure time, % (3 mo)	Normal (0.4%)	Normal (1.6%)

*GERD*, Gastroesophageal reflux disease; *LA*, Los Angeles classification of reflux esophagitis; *POEM-F*, peroral endoscopic myotomy with fundoplication.

The procedure for case 2 lasted 142 minutes (fundoplication 119 minutes). The Eckardt score decreased from 5 to 2 at 12 weeks. Although the patient was asymptomatic, follow-up endoscopy revealed a loose wrap and grade B esophagitis, with normal pH metrics (DeMeester score 9.0, acid exposure 1.6%).

Baseline pH data were unavailable, limiting reflux comparison between the cases.

## Conclusions

These 2 cases demonstrate the technical feasibility of suture-based fundoplication after POEM, offering a potential alternative to traditional loop-ligating methods. Both techniques enabled successful wrap creation using existing endoscopic suturing tools. As devices currently used for POEM-F are off-label and technically demanding, there is growing interest in developing dedicated suturing systems designed to improve procedural efficiency and to be evaluated for their long-term durability.

## Disclosure

The following author disclosed financial relationships: M. Khashab: Consultant for Boston Scientific, Olympus America, Medtronic, and MicroTech; royalties from UpToDate and Elsevier. All other authors disclosed no financial relationships.

## Supplementary data

Video 1 Standard and suture-based techniques for fundoplication in the peroral endoscopic myotomy with fundoplication (POEM-F) procedure.
